# Rare case of endometrial vascular dystrophy: Three case reports

**DOI:** 10.1097/MD.0000000000034546

**Published:** 2023-08-11

**Authors:** JinCheng Huang, WenJian Zhang, Min Guo, Kun Tang, YongJuan Zheng, CuiFen Li

**Affiliations:** a SSL Central Hospital of Dongguan City, Dongguan, Guangdong, China.

**Keywords:** capillary loop, dystrophy, endometrial vascular, histology, hysteroscopy

## Abstract

Endometrial vascular dystrophy refers to abnormal vessels that are very tortuous, dilated, and sometimes thrombosed. Endometrial vascular dystrophy is rare under hysteroscopy.

**Patient:**

All three patients had a history of abnormal uterine bleeding. The duration of vaginal bleeding ranged from 1 month to 2 years. There was no history of unusual diseases, alcohol or drug abuse, or genetic history.

**Diagnoses:**

Endometrial vascular dystrophy.

**Intervention:**

Three patients underwent hysteroscopy and curettage under intravenous general anesthesia. Pathological examination showed secretory endometrium, with one case coexisting with endometrial polyps.

**Outcomes:**

No recurrence was found during postoperative follow-up at 12 months.

**Lessons:**

Endometrial vascular dystrophy is a rare hysteroscopy phenomenon shown in the secretory endometrium. We believe that it was a capillary loop with different manifestations.

## 1. Introduction

Endometrial vascular dystrophy refers to abnormal vessels that are very tortuous, dilated, and sometimes thrombosed.^[1]^ Two types were found. The first was spiral-like vessels evenly distributed on the endometrial surface, and the other was broken meshed or branching capillaries on the superficial plexus. Only a total of 12 cases have been reported up to now.^[2–5]^ And vascular dystrophy is more often reported in patients in the luteal phase of the menstrual cycle. The pathogenesis of endometrial vascular dystrophy is still unknown, but researchers tend to consider it a nonmalignant manifestation. In this study, we show the hysteroscopy characteristics of 3 cases and try to explain how they happened.

## 2. Case report

### 2.1. Case 1

**Figure 1.**
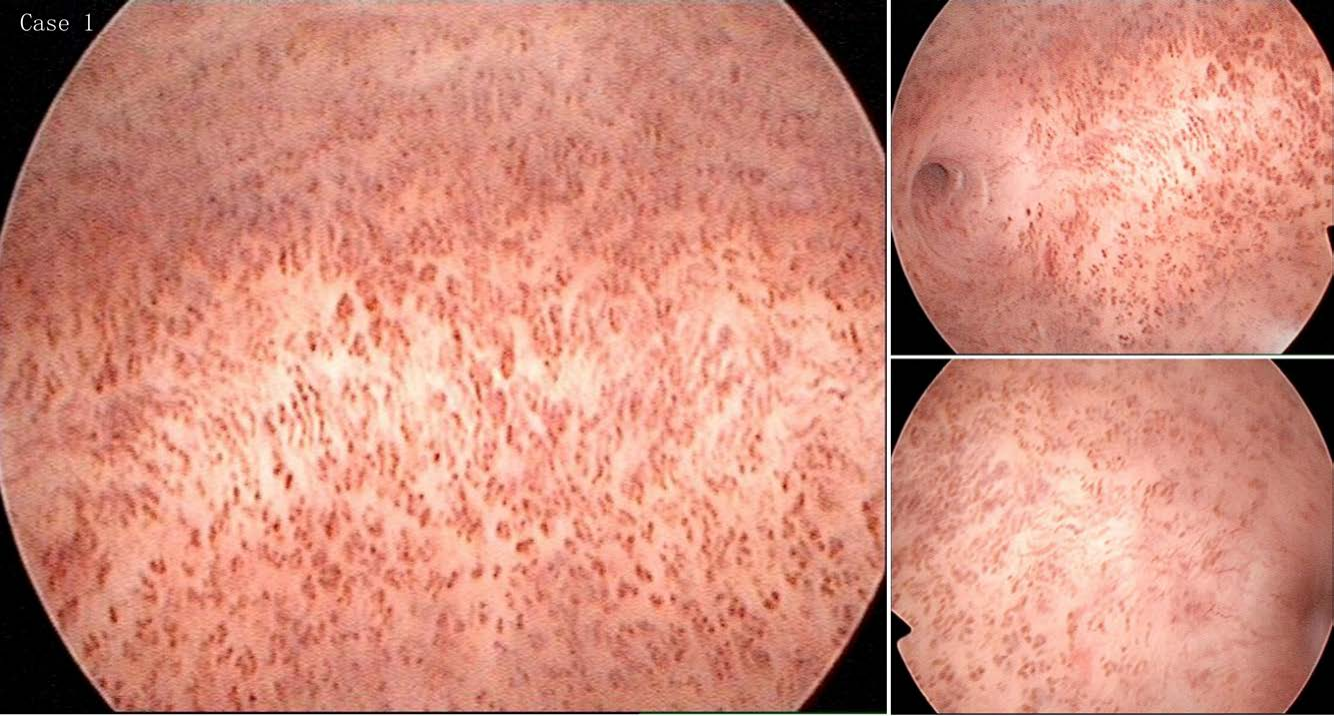
Hysteroscopic image, the endometrium was smooth, and the uterine cavity had a large number of curved and dilated cord-like capillary loops.

A 39-year-old G3P2 woman with slim vaginal bleeding that lasted for 1 month. On pelvic examination, a small amount of blood was seen in the vagina, the cervix was smooth, and the uterus was of normal size. Outpatient hysteroscopy was performed, and hysteroscopy showed endometrial vascular dystrophy. The endometrium was smooth, and the uterine cavity had a large number of curved and dilated cord-like capillary loops (Fig. 1). Perform diagnostic curettage, and pathological findings indicate a secretory phase of the endometrium. Pathological specimens had red blood cells in part of the glands (Fig. 3B), but not in most of them (Fig. 3A). She took combined oral contraceptive pills for 6 months, and no abnormal uterine bleeding was observed.

**Figure 2.**
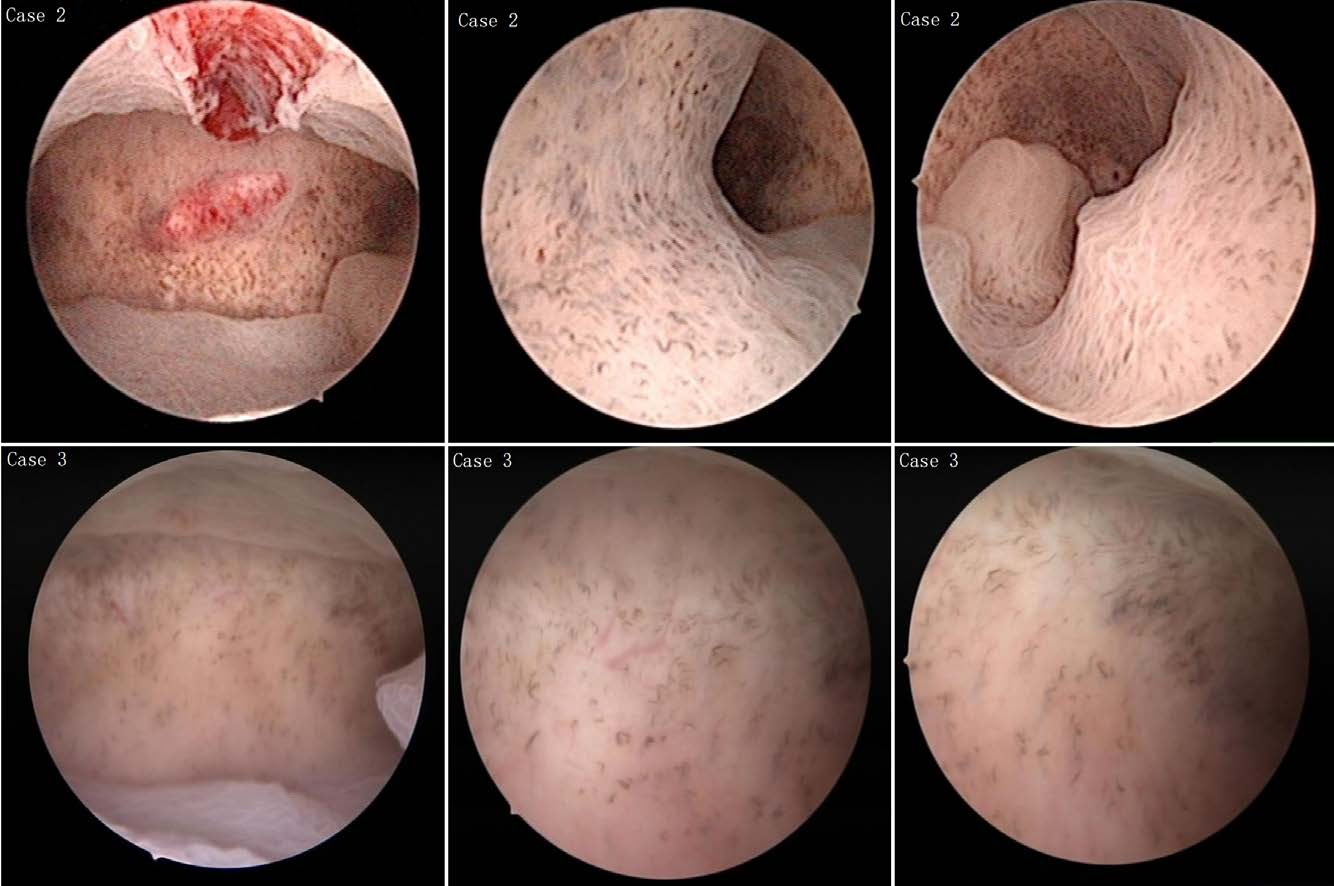
Hysteroscopic image, the endometrium was thickened, the endometrium was grayish white, there was local endometrial polyp formation, and the uterine cavity had a large number of small curved blood vessels.

**Figure 3.**
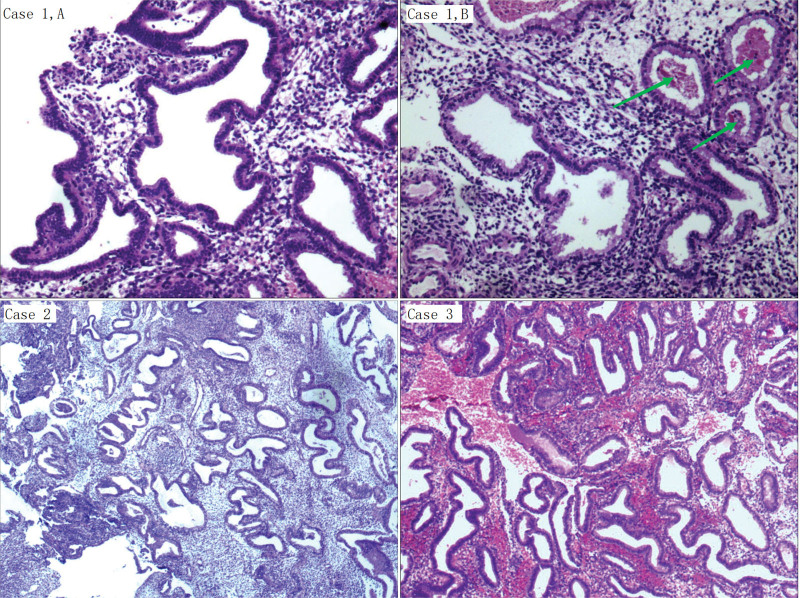
In the pathological section, the arrow in case 1 (B) showed red blood cells in part of the glands, but not in most of them (A); in cases 2 and 3, no blood-filled glands were found in any of the pathological sections.

### 2.2. Case 2

A 43-year-old G3P2 woman, with her last menstrual period 2 weeks ago, presented to the clinic reporting a 6-month history of heavy menstrual bleeding. Her menstrual cycles were every 28 days and lasted 7 days. She bled heavily on days 2–4 with the passage of clots and required changing her feminine pad every 3 hours. Her medical history was remarkable for having had one cesarean section. On pelvic examination, the uterus was enlarged with no adnexal masses. Ultrasound indicated endometrial thickening and uterine fibroids (54 × 49 mm). Hysteroscopy examination indicated endometrial vascular dystrophy. The endometrium was thickened, the endometrium was grayish white, there was local endometrial polyp formation, and the uterine cavity had a large number of curved and dilated cord-like capillary loops (Fig. 2). Perform diagnostic curettage, and pathology showed secretory endometrium with endometrial polyps (Fig. 3). She took combined oral contraceptive pills for 6 months, and no abnormal uterine bleeding was observed.

### 2.3. Case 3

A 43-year-old G2P2 woman complained of heavy vaginal bleeding with clots of 2-year duration. The patient stated that it took 14 to 20 days for her menstruation to be completely clean; the first 4 days were at her usual menstrual volume, and after 4 days, the vaginal bleeding decreased. There was no change in the menstrual cycle. On pelvic examination, a small amount of blood was seen in the vagina, the cervix was smooth, and the uterus was slightly enlarged. Ultrasonography was performed on the 15th day of the menstrual period, and the endometrium was 16 mm and the uterine fibroids in the anterior wall were 36 mm. Hysteroscopy was performed, which suggested endometrial vascular dystrophy. The endometrium was thickened, the endometrium was grayish white, and the uterine cavity had a large number of curved and dilated cord-like capillary loops (Fig. 2). Perform diagnostic curettage, and pathological finding indicated a secretory phase of the endometrium (Fig. 3). No blood-filled glands were found in any of the pathological sections. She took combined oral contraceptive pills for 6 months, and no abnormal uterine bleeding was observed.

Approval for the study protocol was not necessary because our institutional review board does not require approval for case reports. We obtained written informed consent for treatment of the patient.

## 3. Discussion

These curved, dilated capillary loops shown in Figures 1 and 2 can be readily identified. Physical sections provided by Sopelana et al^[4]^ showed glands filled with Periodic Acid Schiff-positive mucopolysaccharides and glycoprotein A-positive blood cells. And he pointed out that these structures were not blood vessels, but glands packed with red blood cells. Unfortunately, the authors are not feasible to explain how the blood gets into the glands. Just as Fernando reported, the structures were glands and filled with blood, which should be seen in all cases. However, this structure was seen only in part of the pathological sections of Case 1, while the other two cases were not found. In the case reported by Jiang and Gong,^[5]^ no blood-filled glands were found by pathology. In our point, these structures were not glands, but highly helical and expansive capillary loop.^[1]^ Remodeling of endometrial blood vessels was related to endometrial differentiation, menstruation, and placenta formation. Angiogenesis has 3 distinct phases in the menstrual cycle. The first stage was repaired by rupturing blood vessels in the menstrual period. The second stage was the rapid growth of the endometrium in the proliferative period. And the third stage was the development of spiral arterioles and the growth of the superficial capillary plexus in secretory phase.^[6]^ The helical arterioles rapidly develop and spiral, exhibiting marked growth during the secretory phase.^[7]^ Red cells were found in the lumen of the endometrial glands in the pathological sections, and the reasons for that may be as follows: on the one hand, the symptom of most of the cases is abnormal uterine bleeding, so it is normal to have mixed blood when taking endometrial specimens. After fixation, only a small number of sections show red blood cells in the glandular ducts. In the video provided by Fernando, a brown fluid similar to that seen in blood vessels is released during hysteroscopy.^[4]^ This brown liquid is precisely the manifestation of hemorrhage under hysteroscopy. On the other hand, if there was blood in the glands, brown fluid will flow out continuously when the dilatation fluid flushes the uterine cavity during hysteroscopy because the opening of the endometrial glands faces the uterine cavity. In addition, due to the compression and folding of the pathological slide, these vessels appear to be located in the glandular cavity. Why do these vessels appear brown or dark red under hysteroscopy? It is likely that the vessels were further bent and dilated under the action of progesterone. The endometrium continues to thicken during the luteal phase, and under certain conditions, the endometrial spiral arteries contract and relaxation rhythmically, which in turn lead to thrombosis of small vessels. This may explain why the cases have abnormal uterine bleeding. Therefore we believe that this most likely relates to progesterone levels.

This vascular image has also been seen in the early diagnosis of esophageal cancer, gastric cancer, and oropharyngeal and hypopharyngeal mucosal sites.^[8–10]^ In particular, with narrow-band imaging, the distribution of capillaries was more clearly visible,^[11,12]^ which improves the accuracy of hysteroscopy diagnosis,^[13,14]^ and it appears to identify endometrial cancer earlier.^[15,16]^ Studies on esophageal gastroscopy in this regard have been very mature, and the esophageal intraepithelial papillary capillary loops have been classified. Inoue summarized the abnormal shapes of capillary loop appear at 4 typical changes as follows: dilation, tortuous weaving, irregular caliber, and form variation.^[17,18]^ Kisu et al^[19]^ described a variety of atypical blood vessels were observed in the mucosa of endometrial malignant lesions, including vessels with expanded, tortuous, and zigzagged shapes; variable diameter; inflectional, interrupted, papillary shapes; and sea anemone shapes. When gynecologists saw these convoluted blood vessels under hysteroscopy, the vast majority of them suspected that the endometrium had malignant lesions that could not be accurately diagnosed at that time. In our study, pathological sections from these patients excluded endometrial malignancy. The inability to diagnose this case may be due to the rarity of endometrial vascular nutrition. As showed in Figures 1 and 2, endometrial vascular dystrophy intima thicknesses and partial density of dilated blood vessels were different among patients.

Fortunately, it was not a malignant lesion, and our patients did not experience any abnormal vaginal bleeding 6 months after treatment. The report showed that complete spontaneous regression of the lesion happened when a second hysteroscopy was carried out in the early proliferative phase of the subsequent menstrual cycle.^[3]^ Because this article is composed of 3 case reports and there are currently very few literature reports, it is prone to bias. The lack of a control group in the 3 case reports may affect the reliability of the conclusions.

## 4. Conclusion

In conclusion, we referred that endometrial vascular dystrophy is a hysteroscopy rare phenomenon shown in the secretory phase. We believe that it was a capillary loop with different manifestations.

## Author contributions

**Conceptualization:** JinCheng Huang, WenJian Zhang, Min Guo, Kun Tang, YongJuan Zheng, CuiFen Li.

**Data curation:** JinCheng Huang.

**Methodology:** Min Guo, Kun Tang, YongJuan Zheng.

**Writing – original draft:** JinCheng Huang, WenJian Zhang.

**Writing – review & editing:** JinCheng Huang, CuiFen Li.
